# The duration of postoperative analgesic use after total knee arthroplasty and nomogram for predicting prolonged analgesic use

**DOI:** 10.3389/fsurg.2022.911864

**Published:** 2022-07-26

**Authors:** Yi Zhang, Zihua Li, Qihang Su, Heng’an Ge, Biao Cheng, Meimei Tian

**Affiliations:** ^1^ Department of Orthopaedics, Shanghai Tenth People's Hospital, School of Medicine, Tongji University, Shanghai, China; ^2^ Department of Orthopaedics, Shanghai Tongji Hospital, Tongji University School of Medicine, Shanghai, China

**Keywords:** Arthroplasty, analgesic use, NSAID, postsurgical pain, nomogram

## Abstract

**Background:**

Total knee arthroplasty is currently a reliable treatment for end-stage knee osteoarthritis. However, chronic postsurgical pain (CPSP) is substantially thought to reduce patient satisfaction. NSAID-based oral analgesics were used to manage CPSP, but research on the duration of postoperative analgesic use (DAU) and prolonged analgesic use (PAU) are presently scarce.

**Methods:**

Preoperative, perioperative, and one-year or above postoperative follow-up data were collected from 162 patients who underwent total knee arthroplasty between 1 June 2018 and 1 March 2019, and the DAU and the discontinuation time of each patient after discharge were recorded. Observational statistical analysis, diagnostic test, and predictive nomogram construction were performed on the collected data.

**Results:**

The 3-month DAU has good diagnostic utility for poor outcome of postoperative months twelve (POM12). The constructed nomogram shows that gender, preoperative Numeric Rating Scale (NRS) movement pain scores, duration of surgery, postoperative days three (POD3) moderate to severe movement pain, and POD3 pain rescue medication were significant prognostic predictors of PAU after discharge. The area under the curve (AUC) of the 3-month, 6-month, and 12-month nomogram receiver operating characteristic (ROC) curves were calculated to be 0.741, 0.736, and 0.781.

**Conclusion:**

PAU was defined as more than three months of NSAID-based oral analgesic use after TKA. Prognostic predictors of PAU after TKA were identified, and visualized nomogram was plotted and evaluated. The evaluation indicated that the prediction model had the good predictive ability and was a valuable tool for predicting PAU after discharge.

## Introduction

Knee osteoarthritis (OA), the most common degenerative musculoskeletal disorder, is a significant factor responsible for increasing years of living with disability (YLDs) (1). Although total knee arthroplasty (TKA) is currently a reliable treatment for end-stage osteoarthritis patients with pain and disability, 11% to 18% of postoperative patients are dissatisfied with the procedure (2, 3). Chronic postsurgical pain (CPSP), defined as pain lasting for more than three months after surgery, is regarded as a critical factor of postoperative dissatisfaction (3–5). Although CPSP has attracted much attention in the academic community, prolonged analgesic use (PAU), which often accompanies CPSP, has rarely been studied.

Oral analgesics, playing a pivotal role in the management method of CPSP, can lead to adverse consequences if being overused for a long time (whether opioid or nonopioid) (6–8). In the context of the opioid epidemic in the United States, most studies have focused on short-term postoperative opioid consumption (8–11). However, few studies have paid attention to the long-term use of NSAID-based oral analgesics for CPSP after discharge. To date, little is known about NSAID-based duration of postoperative analgesic use (DAU), and it is not clear what factor is associated with DAU. Identifying risk factors for long-term postoperative pain medication use will help surgeons distinguish patients with a high risk of prolonged analgesic use and develop a proper intervention protocol to reduce analgesic consumption.

Based on the results of this study, PAU was defined as more than three months of NSAID-based oral analgesic use after TKA. The aim of this study is to explore the situation of analgesic use in patients after TKA and evaluate factors that may be associated with PAU. It is hypothesized that the DAU is closely associated with prognosis and can be predicted by multiple variables.

## Materials and methods

### Patients selection

This study was approved by the Ethics Committee of the hospital (SHSY-IEC-4.1/20-21/01), and adhered to the tenets of the Declaration of Helsinki. Informed consents were obtained from all participants enrolled in the study. The study collected retrospective data from patients who underwent TKA between 1 June 2018 and 1 March 2019. Baseline demographics, patient preoperative characteristics, perioperative treatment details, one-year or above postoperative follow-up outcomes, and several other factors were collected and analyzed subsequently.

Inclusion criteria: (1) aged over 50 years old; (2) elective total knee arthroplasty (include primary surgery and revision); (3) no history of joint infection. Exclusion criteria: (1) periarticular tumor; (2) TKA combined with other operations simultaneously; (3) incomplete data and lack of one or more laboratory indicators.

### Perioperative and postoperative protocols

All surgeries were performed separately by five senior surgeons in the hospital *via* medial parapatellar approach under a tourniquet. All surgical patients were given general anesthesia, and additional ultrasound-guided femoral nerve block was provided to a subset of patients at the anesthesiologist's discretion based on patient condition. At the end of the surgery, 150 mg ropivacaine, 7 mg compound betamethasone, and 1 g tranexamic acid were mixed as a 50 ml injection solution and used for periarticular local Infiltration. Patients received a 48-hour patient-controlled intravenous anesthesia (PCIA) consisting of a continuous intravenous infusion (butorphanol 0.12 mg/ml with dexmedetomidine 1 µg/ml at a rate of 2 ml/h) and patient-activated bolus doses of 0.5 ml of the solution with a 15-min lockout period after each activation.

After returning to the ward, patients were transitioned to multimodal pain management (MMPM) consisting of morphine sulfate 10 mg and ketorolac 60 mg every 12 h and gabapentin 0.3 g *tid*, and cryotherapy was performed around the surgical incision. Breakthrough pain does not react to the above protocol was managed by rescue medication consisting of pethidine 100 mg plus promethazine 25 mg. All patients received and implemented perioperative and postoperative rehabilitation training according to clinical guidelines (12).

After discharge, the analgesic prescription was filled and refilled by the attending doctor according to the World Health Organization Three-Step Analgesic Ladder. All patients were instructed to use analgesic medications only as needed after discharge and to stop the medication when the pain was relieved to easily tolerable mild pain. Levels of pain were recorded utilizing the 11-point Numeric Rating Scale (NRS: 0 no pain, 10 worst pain imaginable). Furthermore, postoperative pain greater than four was defined as moderate to severe pain.

### Follow-up and data extraction

All patients were recommended to receive regular follow-up visits after discharge. Patients were generally followed up at 1, 3, 6, and 12 months for the first year and every six months thereafter. Patients' satisfaction, Lysholm score, and rest/movement pain were evaluated and recorded at postoperative months twelve (POM12) follow-up. Cases that have the following four conditions at POM12 were defined as POM12 poor outcome: (1) POM12 satisfaction score <50; (2) POM12 Lysholm score <70; (3) POM12 moderate to severe rest pain; (4) POM12 moderate to severe movement pain. Cases with the same one of the above conditions were considered subgroups of POM12 poor outcomes.

Data such as patient characteristics, comorbid conditions, laboratory indicators, information about surgery, and immediate outcomes during hospitalization were collected from medical records. The follow-up data were obtained from the outpatient records, and further information was obtained from the telephone follow-up if needed. The data extraction was implemented by two researchers (MMT, ZHL) manually.

### Statistical analysis

The obtained data are summarized and classified into measurement data (including continuous and discrete variables) and categorical data (including ordinal and unordered categorical variables). Descriptive statistics were performed on the collected data. To assess the clinical implications of DAU, a diagnostic test evaluation using DAU to diagnose adverse prognosis was performed.

In the next step of prediction model construction, the preoperative and perioperative predictor variables were all included in the univariate analysis of the Cox proportional hazards regression model at first. Endpoint events were defined as cessation of analgesic use after discharge and no or only slight tolerable pain at this time. Based on Akaike information criteria (AIC) and Bayesian information criterion (BIC), potential prognostic factors (*p* < 0.10) in the univariate analysis were screened with stepwise regression and included in the multivariate Cox regression analysis. Kaplan–Meier survival analysis was performed to further analyze categorical variables in prognostic factors, and nonparametric group comparisons were performed using the log-rank test. Moreover, a predictive nomogram model was constructed using the results of the Cox multivariate analysis to visualize the predicting of the 3-month, 6-month, and 12-month PAU. The predictive power of the nomogram was verified by plotting receiver operating characteristic (ROC) curves and calibration curves. The model performance for predicting results was further evaluated by calculating Harrell's concordance index (C-index), and the internal verification was carried out using 1,000 sets of bootstrap sampling.

Statistical analyses were performed with R (version 4.1.1, The R Foundation), including the “survival” and “rms” packages. *p* < 0.05 was considered to be statistically significant.

## Results

### Screening process and cohort characteristics

**Figure 1:**
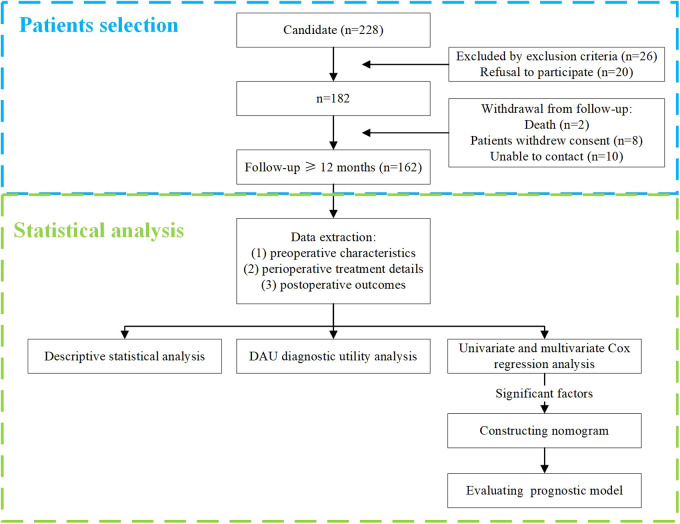
The flow chart of research process.

A total of 182 patients met the inclusion criteria and were eligible for the study, and 20 were excluded for withdrawing from follow-up. Therefore, the final cohort consisted of 162 patients with at least one year of postoperative follow-up, and their discontinuation time and clinical data were recorded. The flow chart of the study process, including patient selection details, is illustrated in Figure 1. Patient characteristics and categorical data were presented in Tables 1, 2, and included in subsequent statistical analyses.

**Table 1:** Patients’ characteristics (measurement data).

Characteristics	Mean	SD
Age (years)	68.37	6.652
BMI (kg/m^2^)	26.27	3.997
Preoperative NRS rest pain scores	0.27	0.856
Preoperative NRS movement pain scores	4.12	1.385
Duration of surgery (minutes)	84.54	22.457
Preoperative blood tests		
Preoperative CRP (mg/l)	4.08	6.295
Preoperative RBC (10^12^/l)	4.42	0.514
Preoperative HCT (vol%)	39.67	4.107
Preoperative lymphocyte (10^9^/l)	1.84	0.600
Preoperative HB (g/l)	132.56	12.271
Preoperative neutrophil (10^9^/l)	3.90	1.428
Preoperative NLR	4.08	6.294
POD1 blood test		
POD1 CRP (mg/l)	36.04	31.141
POD1 RBC (10^12^/l)	3.92	0.460
POD1 HCT (vol%)	34.28	5.509
POD1 lymphocyte (10^9^/l)	1.00	0.405
POD1 HB (g/l)	118.47	13.656
POD1 neutrophil (10^9^/l)	10.26	2.692
POD1 NLR	11.91	5.687
Duration of hospital stay	9.47	3.211
POM12 Lysholm score	81.90	17.357
POM12 satisfaction	85.50	21.987
Duration of postoperative analgesic use (months)	2.65	4.373

NRS, Numeric Rating Scale; CRP, C-reactive protein; RBC, red blood cell count; HCT, hematocrit; HB, hemoglobin; NLR, neutrophil-to-lymphocyte ratio; POD3, postoperative days three; POM12, postoperative months twelve.

**Table 2:** Patients’ characteristics (categorical data).

Characteristics	Category	*n*	Percent (%)
Age (years)	80–89	14	8.6
	70–79	56	34.6
	60–69	76	46.9
	50–59	16	9.9
Gender	Male/ Female	28/134	17.3/82.7
BMI (kg/m^2^)	≥30.00 (obesity)	24	14.8
	25.00–29.99 (overweight)	71	43.8
	18.5–24.99 (normal weight)	64	39.5
	<18.5 (underweight)	3	1.9
ASA grade	≤2/≥3	147/15	90.7/9.2
Diagnosis	OA	153	94.4
	Post-traumatic OA	4	3.1
	Rheumatoid arthritis	5	2.5
Presurgical duration of pain (months)	>6	125	77.2
	3–6	9	5.6
	1–3	13	8.0
	<1	15	9.3
Hypertension	+/−	106/56	65.4/34.6
Diabetes	+/−	38/124	23.5/76.5
Prosthetic type	PS/CR	114/48	70.4/29.6
Operator	A	42	25.9
	B	5	3.1
	C	84	51.9
	D	27	16.7
	E	4	2.5
Side	Left	78	48.1
	Right	79	48.8
	Both	5	3.1
Primary knee arthroplasty	primary/revisionary	139/23	85.8/14.2
Femoral nerve block	Yes/no	145/17	89.5/10.5
Duration of surgery (minutes)	≥120	9	5.6
	90–119	58	35.8
	60–89	71	43.8
	<60	24	14.8
Indwelling drainage	Yes/no	80/82	49.4/50.6
PCIA	Yes/no	65/97	40.1/59.9
POD3 moderate to severe rest pain	+/−	18/144	11.1/88.9
POD3 moderate to severe movement pain	+/−	112/50	69.1/30.9
POD3 pain rescue medication	+/−	16/146	9.9/90.1
POD1 PONV	+/−	43/119	26.5/73.5
Transferred to a rehabilitation hospital after discharge	Yes/no	6/156	3.7/96.3
POM12 satisfaction score	<50/≥50	29/133	17.9/82.1
POM12 Lysholm score	<70/≥70	13/149	8.0/92.0
POM12 moderate to severe rest pain	+/−	7/155	4.3/95.7
POM12 moderate to severe movement pain	+/−	28/134	17.3/82.7
POM12 poor outcome	+/−	41/121	25.3/74.7
PAU	+/−	40/122	24.7/75.3

BMI, body mass index; ASA, the American Society of Anesthesiologists; PCIA, patient-controlled intravenous anesthesia; PONV, postoperative nausea/vomiting; NRS, Numeric Rating Scale; CRP, C-reactive protein; RBC, red blood cell count; HCT, hematocrit; HB, hemoglobin; NLR, neutrophil-to-lymphocyte ratio; POD3, postoperative days three; POM12, postoperative months twelve; PAU, prolonged analgesic use.

### Observation of clinical outcomes

**Figure 2:**
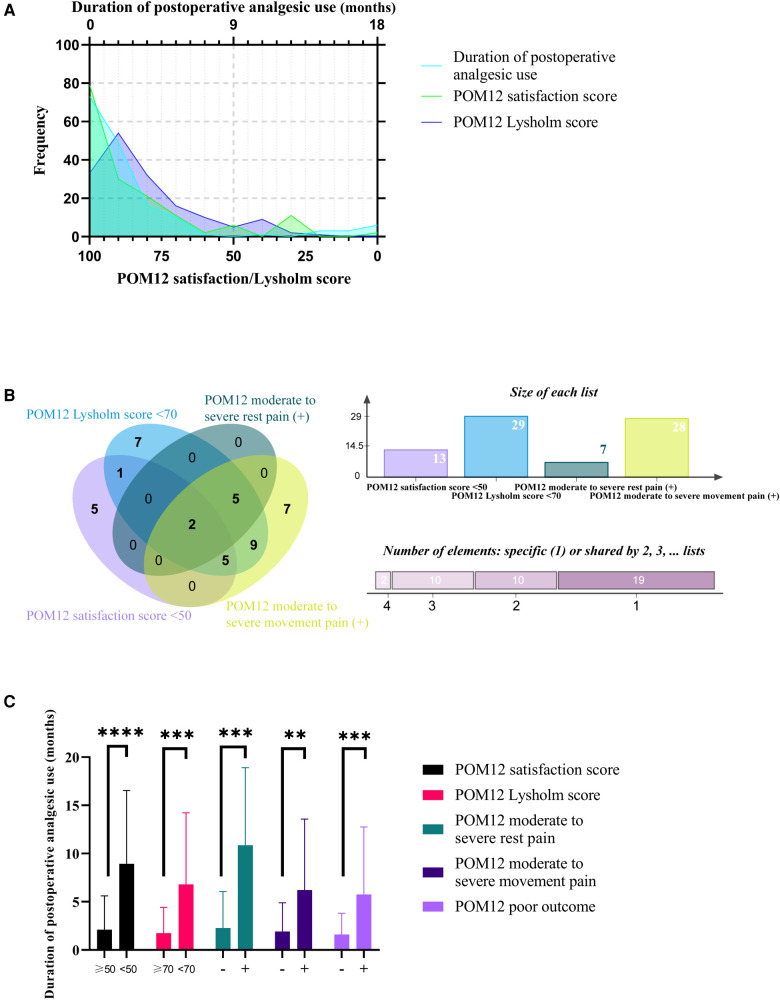
Descriptive statistical analysis of DAU. (**A**) Frequency distribution of DAU, POM12 satisfaction score, and POM12 Lysholm score. (**B**) Venn diagram of four subgroups of POM12 poor outcome. (**C**) DAU of cases with or without adverse outcomes. ***, *p* < 0.001; ****, *p* < 0.0001.

The preliminary descriptive statistical analysis was performed to display the characteristics of DAU's data. The normality test showed that DAU, POM12 satisfaction score, and POM12 Lysholm score were non-normal distribution (Figure 2A). The correlation between DAU and the other two continuous variables was tested using the Spearman method, respectively. The results of the correlation analysis show that both correlation coefficients were less than 0.3, although both significance levels were less than 0.001. The distribution of the subgroups of POM12 poor outcome is shown in Figure 2B, indicating heterogeneity within the poor outcome population. One remarkable result is that the *t*-test indicates that the DAU of cases with adverse outcomes were significantly longer than their counterpart (Figure 2C). These results suggest that DAU and POM12 poor outcomes are closely related.

### Diagnostic utility of DAU

**Figure 3:**
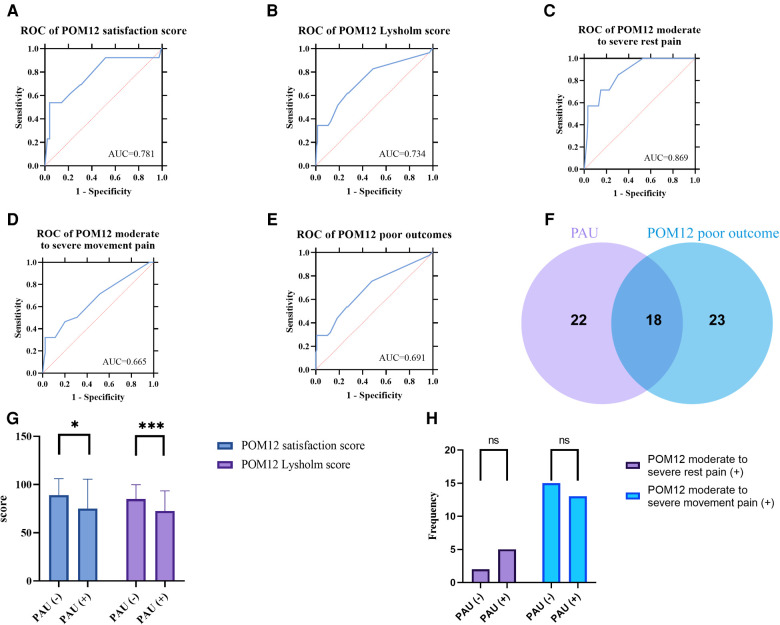
(**A–E**) ROC curve of DAU for different POM12 poor outcomes. (**F**) Venn diagram of PAU and POM12 poor outcome. (**G**) POM12 satisfaction score and POM12 Lysholm score of cases with or without PAU. (**H**) Frequency distribution histogram of POM moderate to severe pain.

In order to define PAU, a diagnostic test evaluation using DAU as a diagnostic method for adverse prognosis was implemented. As shown in Figures 3A–E, The ROC curves plotted for the poor outcome and its four subgroups all demonstrate that DAU has a good diagnostic utility for prognosis (AUC: 0.781, 0.734, 0.869, 0.665, and 0.691). To further determine the cut-off value, we list the Youden index of each ROC curve in Table 3. Although the cut-off value of >2.5 (i.e. three months) ranks third, it has better practical value than the top two whose values are too large (>12 and >9).

**Table 3:** Youden index of ROC curves of DAU (sorted by ∑ Youden index in descending order).

Cut-off Value	Youden index					∑ Youden index
	POM12 satisfaction	POM12 Lysholm score	POM12 moderate to severe rest pain	POM12 moderate to severe movement pain	POM12 poor outcome	
>12.00	0.4212	0.3298	0.5198	0.299	0.2572	1.827
>9.000	0.4982	0.3222	0.5133	0.2915	0.1463	1.7715
>2.500	0.4006	0.3292	0.4885	0.2628	0.2844	1.7655
>1.750	0.3903	0.35	0.5474	0.2015	0.2721	1.7613
>7.000	0.4915	0.3147	0.5069	0.2841	0.1624	1.7596
>1.250	0.3836	0.3425	0.541	0.194	0.2639	1.725
>3.500	0.3976	0.2515	0.5659	0.2228	0.26	1.6978
>0.7500	0.4063	0.3389	0.471	0.1994	0.2768	1.6924
>14.50	0.3443	0.2953	0.5262	0.2633	0.1931	1.6222
>4.500	0.4244	0.2395	0.4424	0.2095	0.2356	1.5514
>5.500	0.4311	0.2471	0.4488	0.2169	0.1868	1.5307
>15.50	0.1905	0.2264	0.5391	0.1919	0.1935	1.3414
>16.50	0.1972	0.1919	0.3963	0.1562	0.2018	1.1434
>17.50	0.2107	0.1649	0.2599	0.128	0.2762	1.0397
>0.1750	0.006711	0.007519	0.006452	0.007463	0.00826	0.036409
>0.3750	−0.05005	−0.00442	0.03226	0.03731	0.00866	0.02376

One interesting result was that there are only about half elements of PAU belong to POM12 poor outcomes (Figure 3F), suggesting that PAU is significantly different from POM12 poor outcomes. By comparing groups with and without PAU, it was found that POM12 satisfaction and Lysholm score were significantly lower in the PAU group, while the incidences of moderate to severe pain were not significantly different (The chi-square test *p*-value > 0.05) (Figures 3G,H).

### Univariate and multivariate regression analyses

**Figure 4:**
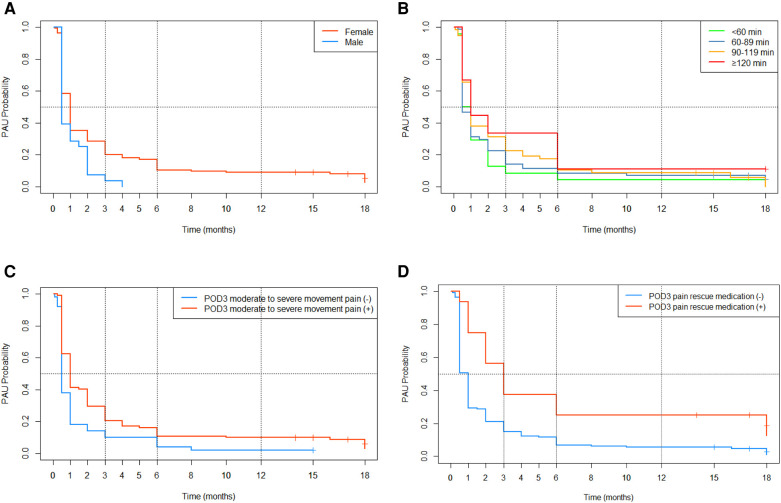
K-M survival curves for categorical data. (**A**) Gender; (**B**) duration of surgery; (**C**) POD3 moderate to severe movement pain (**D**) POD3 pain rescue medication.

As Table 4 shows, history of arthralgia and POD3 moderate to severe rest pain, although *p*-values < 0.10 in the univariate analysis, were dropped from the multivariate fitting model *via* the stepwise regression method. With the lower AIC (1284.489) and BIC (1299.706) value, the model including five predictors (gender, preoperative NRS movement pain scores, duration of surgery, POD3 moderate to severe movement pain, and POD3 pain rescue medication) is the better predictive model. The K-M survival curves for categorical data were plotted and shown in Figures 4A–D, and there are statistically significant differences between all groups.

**Table 4:** Cox proportional hazards regression model of PAU.

	Factors	Univariate analysis			Multivariate analysis		
		HR	95%CI	*p* value	HR	95%CI	*p* value
**Characteristics**	**Age** (80–89/70–79/60–69/50–59 years)	0.9	0.74–1.09	0.282			* *
	**Gender** (male/female)	1.7	1.12–2.58	0.013	1.62	1.06–2.47	*0*.*026*
	**BMI** (obesity/overweight/normal weight/underweight)	0.9	0.72–1.12	0.341			* *
	**ASA grade** (≤2/≥3)	0.9	0.53–1.53	0.694			* *
	**Diagnosis** (OAas reference)	1		* *			* *
	Post-traumatic OA	1.86	0.64–3.86	0.226			* *
	Rheumatoid arthritis	1.58	0.68–5.05	0.319			* *
	**Presurgical duration of pain** (>6/3–6/1–3/<1)	0.86	0.73–1.01	0.061			*NS*
	Preoperative NRS **rest pain** scores	1.05	0.87–1.27	0.601			* *
	Preoperative NRS **movement pain** scores	0.88	0.79–0.99	0.028	0.88	0.79–0.98	*0*.*022*
	**Hypertension** (yes/no)	1.09	0.78–1.52	0.615			* *
	**Diabetes** (yes/no)	0.89	0.61–1.29	0.524			* *
	Preoperative **systemic disorders**	0.96	0.82–1.13	0.631			* *
	Preoperative **CRP**	1.01	0.98–1.04	0.454			* *
	Preoperative **RBC**	1.03	0.76–1.39	0.856			* *
	Preoperative **HCT**	1.03	0.99–1.08	0.159			* *
	Preoperative **lymphocyte**	0.96	0.73–1.27	0.795			* *
	Preoperative **HB**	1.01	1.00–1.02	0.194			* *
	Preoperative **neutrophil**	1.03	0.90–1.16	0.698			* *
	Preoperative **NLR**	1.02	0.87–1.21	0.785			* *
**Operative data**	**Operator** (Aas reference)	1		* *			* *
	B	0.61	0.24–1.55	0.296			* *
	C	0.82	0.56–1.20	0.297			* *
	D	0.69	0.41–1.15	0.154			* *
	E	1.12	0.40–3.14	0.825			* *
	**Side** (Left as reference)	1					* *
	Right	0.96	0.69–1.32	0.782			* *
	Both	0.47	0.17–1.30	0.145			* *
	**Prosthetic type** (PS/CR)	0.92	0.87–0.97	0.152			* *
	**Primary** knee arthroplasty (primary/revisionary)	1.36	0.86–2.16	0.188			* *
	**Femoral nerve block** (yes/no)	1.16	0.70–1.92	0.565			* *
	**Duration of surgery** (≥120/90–119/60–89/<60)	0.83	0.68–1.01	0.060	0.75	0.61–0.92	*0*.*006*
	**Indwelling drainage** (yes/no)	1.14	0.83–1.56	0.421			* *
**Postoperative data**	**PCIA**	1.06	0.77–1.47	0.714			* *
	POD3 **moderate to severe rest pain**	0.43	0.25–0.73	0.002			*NS*
	POD3 **moderate to severe movement pain**	0.59	0.42–0.83	0.002	0.65	0.46–0.93	*0*.*018*
	POD3 **pain rescue medication**	0.41	0.23–0.73	0.003	0.43	0.24–0.78	*0*.*005*
	POD1 **PONV**	0.78	0.54–1.12	0.178			* *
	POD1 **CRP**	1.00	1.00–1.01	0.477			* *
	POD1 **RBC**	1.04	0.74–1.46	0.821			* *
	POD1 **HCT**	1.00	0.97–1.03	0.985			* *
	POD1 **lymphocyte**	1.06	0.71–1.58	0.781			* *
	POD1 **HB**	1.01	0.99–1.02	0.399			* *
	POD1 **neutrophil**	1.00	0.95–1.06	0.866			* *
	POD1 **NLR**	1.00	0.97–1.03	0.906			* *
	**Duration of hospital stay**	0.96	0.91–1.01	0.160			* *
	Transferred to a **rehabilitation hospital** after discharge	0.55	0.24–1.25	0.151			* *

BMI, body mass index; ASA, the American Society of Anesthesiologists; PCIA, patient-controlled intravenous anesthesia; PONV, postoperative nausea/vomiting; NRS, Numeric Rating Scale; CRP, C-reactive protein; RBC, red blood cell count; HCT, hematocrit; HB, hemoglobin; NLR, neutrophil-to-lymphocyte ratio; PCIA, patient-controlled intravenous anesthesia; POD3, postoperative days three; POM12, postoperative months twelve.

### Nomogram of the prognostic model and evaluation of its predictive accuracy

**Figure 5:**
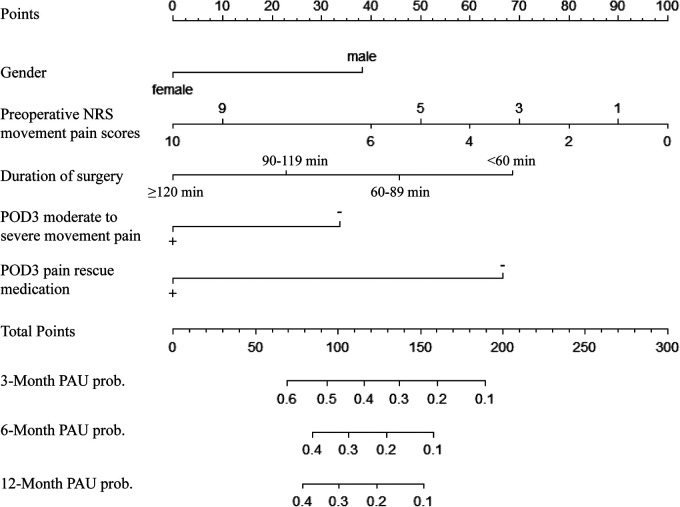
Nomogram for predicting PAU.

**Figure 6:**
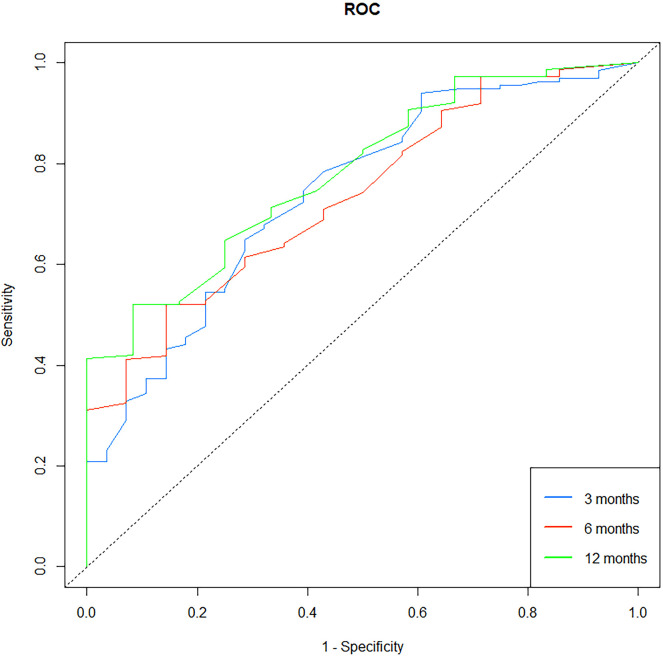
Receiver operating characteristic (ROC) curve for the prediction model. The 3-month, 6-month, and 12-month areas under the curve (AUC) were 0.741, 0.736, and 0.781.

**Figure 7:**
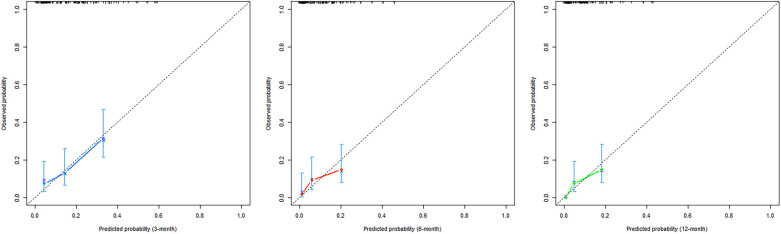
Calibration curves of the nomogram. The x-axis shows the predicted probability of PAU, and the y-axis shows the observed probability of PAU.

A nomogram was built using the results of the multivariable analysis to predict prognostic outcomes visually (Figure 5). The ROC curves and calibration curves were plotted using R to identify the model's predictive ability (Figures 6, 7). The area under the curve (AUC) of the 3-month, 6-month, and 12-month ROC curves were calculated to be 0.741, 0.736, and 0.781, which showed the good predictive ability of the present prediction model. The calibration curves with a good fit between the model-estimated probability and the actually observed probability further validate the model's accuracy. The calculated original C-index for the nomogram was 0.680, and the corrected C-index by bootstrap validation (1,000 bootstrap samples) was 0.666.

## Discussion

In reviewing the literature, no data was found about the situation of the duration of NSAID-based analgesic use after TKA. One of the aims of this study was to investigate the DAU after TKA and to define PAU. The current study found that the DAU of patients who underwent TKA is non-normally distributed (Figure 2A). The DAUs of cases with adverse outcomes were all significantly longer than their counterparts (Figure 2C). The results of diagnostic test evaluation (Figures 3A–E) demonstrate that DAU has good predictive utility for POM12 clinical outcomes. One interesting result was that the cut-off value of >2.5 had a maximum validity. It is desirable to diagnose and predict patient status in the clinic as soon as possible. However, the top two cut-off values (>9 and >12) are too large to make a timely diagnosis of PAU, and they have little significance for the POM12 outcome. Therefore, this study proposes to define PAU as more than three months of NSAID-based oral analgesic use after TKA. This time threshold also accords with CPSP, defined as the pain that persists for more than three months following surgical intervention and where additional particular neuropathic symptoms are observed (13). There are similarities and even possible multicollinearity between PAU and CPSP, but it has been documented that postoperative pain and analgesic consumption remain different (14). Another important result was that there are only about half elements of both sets of PAU and POM12 poor outcomes in the intersection, respectively (Figure 3F). Contrary to expectations, this study did not find a significant difference in the incidence of POM12 moderate to severe pain between cases with and without PAU, although both POM12 satisfaction and Lysholm score were significantly lower in the PAU group (Figures 3G,H). A possible explanation for this might be that other factors, including psychiatric and psychological factors, contribute to the patient's inability to discontinue the medication autonomously or unwillingness to use analgesics continuously. Another possible explanation is that there may be a statistical error caused by the small sample size of this study. These findings suggest that PAU may be able to be a new indicator of TKA's poor outcome. Future large sample studies, which dynamically observe changes in DAU and patient pain profiles and record the reasons for discontinuation and non-discontinuation, will need to be undertaken.

Another aim of the current research was to find the predictors of PAU. Preoperative NRS movement pain scores and POD3 moderate to severe movement pain partially represent preoperative pain and APSP. A strong relationship between acute postsurgical pain (APSP) or preoperative pain and CPSP had been extensively validated (15–21). However, few studies have discussed rest-pain (RP) and movement-pain (MP) separately (15, 19, 21–23). In the current study, MP plays a more critical role than RP, both preoperatively and postoperatively. This finding supports the structural equation modeling proposed by Sayers (17) that chronic pain after TKA was mainly correlated with the severity of MP. For the moment, differentiating RP and MP is gaining increasing attention in the field of surgical research, for the two are mediated by different mechanistic pathways and have different clinical implications (17, 24–26). In OA, MP usually appears more pronounced and earlier than RP and can be significantly relieved after rest (26). Additionally, MP is also a major barrier to good postoperative rehabilitation. Therefore, it has been argued that the assessment and intervention for MP are more critical than RP, and the different biological mechanisms underlying the two types of pain and pharmacological strategies have been investigated (24–27, 28).

Previous studies have demonstrated that patients' preoperative pain sensitivity correlated with MP but not with RP (25, 29, 30). Among these studies, Fassoulaki et al. (30) showed that gabapentin, an anti-hyperalgesic agent, can significantly alleviate MP and postoperative analgesic requirements. Although in the study of Fassoulaki, chronic pain, except for burning pain, was unaffected by the gabapentin, the reason may be that patients received only the short-term gabapentin treatment (10 days). Therefore, it can be assumed that anti-hyperalgesic treatment represented by gabapentin can eliminate PAU by relieving MP. Future studies, which aim to validate this hypothesis into account, will need to be undertaken.

One unexpected finding was that the longer operative time was predictive of PAU, while the different operators did not influence the PAU. This finding is inconsistent with previous studies, which demonstrated that the duration of arthroplasty did not affect postoperative pain and patient satisfaction (31). The duration of surgery is an indicator affected by the surgeon's experience and several other factors, including BMI, gender, ASA, etc. (31–33). However, in the current study, the five chief surgeons were all experienced doctors who had reached a plateau after the learning curve. Hence, a possible explanation for this finding might be that individual surgical complexity (rather than the similarly skilled surgeons) contributed to PAU while causing a prolonged duration of surgery. Longer operative time may suggest more surgical trauma and more variability in operator manipulation. Previous studies have demonstrated that longer operative time is associated with the development of postoperative complications. The sample size of several surgeon subgroups may be too small to show significant differences between groups. This issue can be verified by future studies with larger samples. This study provides further evidence for recommending minimizing operation times, without compromising the technical components of the surgical procedure, to improve surgical outcomes following TKA.

Another finding of the current study is that although intraoperative nerve block, PCIA, and rescue medication (RM) are all optional components in the MMPM, nerve block and PCIA have no significant effects on PAU development. Unlike nerve block and PCA (which are primarily at the physician's discretion), whether RM is administered or not depends on the patient's subjective reported pain intensity. On the one hand, a possible explanation for the significant association of RM with PAU is the psychological factors. Psychological factors represented by catastrophizing amplified the patient-reported pain intensity, promoting RA use and further prolonged analgesia use, which means that fear of pain leads to anticipation and dependence on analgesics. Therefore, it can be assumed that appropriate patient education and psychological interventions for postoperative arthroplasty patients can influence patient analgesic use and pain. Future studies on the current topic are recommended. On the other hand, RA did represent patients' acute postoperative pain intensity, so the occurrence of breakthrough pain postoperatively was significantly associated with PAU. Future research should investigate the mechanisms underlying acute postsurgical pain, including breakthrough pain, to develop novel targets and therapies to prevent PAU.

A limitation of this study is that the restricted sample size causes some categorical data-based classifications to be too small and may bias the statistical results. Future studies with larger sample sizes need to be carried out. Secondly, because of the limitation of retrospective study, the patients were not tested for some inflammatory markers at the time. The relationship between inflammatory markers and PAU is worthy exploring in the future.

## Conclusions

The present study was designed to explore the situation of analgesic use in patients after TKA and evaluate factors that may be associated with PAU. Based on the diagnostic test results, PAU was defined as more than three months of NSAID-based oral analgesic use after TKA. Moreover, the Cox proportional hazards regression model results reveal that gender, preoperative NRS movement pain scores, duration of surgery, POD3 moderate to severe movement pain, and POD3 pain rescue medication were prognostic predictors of PAU after TKA. A visualized nomogram was plotted according to the results of multivariable analysis, and the model evaluation demonstrated that it had a good predictive ability. The current study is the first to investigate the long-term use of NSAID-based oral analgesics for CPSP after discharge, and the specific mechanisms of PAU and pain remain to be determined.

## Data Availability

The raw data supporting the conclusions of this article will be made available by the authors, without undue reservation.

## Employment

All authors certify that they have no affiliations with or involvement in any organization or entity with any financial interest or non-financial interest in the subject matter or materials discussed in this manuscript.
